# Measurement of Practices-Knowledge-Attitudes of the Nursing Process: Systematic Review

**DOI:** 10.17533/udea.iee.v39n3e15

**Published:** 2021-11-08

**Authors:** Fabio Alberto Camargo-Figuera, María Alejandra Ortega-Barco, María Camila Rojas-Plata, Daniela Marín-Rodríguez, Lizeth Johana Alarcón-Meléndez, Beatriz Villamizar-Carvajal

**Affiliations:** 1 Nurse, PhD. Professor. Universidad Industrial de Santander, Colombia Email: falcamfi@uis.edu.co Universidad Industrial de Santander Universidad Industrial de Santander Colombia falcamfi@uis.edu.co; 2 Nurse, Master’s. Professor. Universidad Industrial de Santander, Colombia Email: maorteba@correo.uis.edu.co Universidad Industrial de Santander Universidad Industrial de Santander Colombia maorteba@correo.uis.edu.co; 3 Nursing student, COLCIENCIAS Young Researcher. Universidad Industrial de Santander, Colombia Email: camilarojas9904@gmail.com Universidad Industrial de Santander Universidad Industrial de Santander Colombia camilarojas9904@gmail.com; 4 Nurse, COLCIENCIAS Young Researcher. Universidad Industrial de Santander, Colombia Email: danymarinr@gmail.com. Corresponding author. Universidad Industrial de Santander Universidad Industrial de Santander Colombia danymarinr@gmail.com; 5 Nurse, Specialist. Universidad Industrial de Santander, Colombia Email: lizalarcon33@gmail.com Universidad Industrial de Santander Universidad Industrial de Santander Colombia lizalarcon33@gmail.com; 6 Nurse, PhD. Professor. Universidad Industrial de Santander, Colombia Email: beatriz@uis.edu.co Universidad Industrial de Santander Universidad Industrial de Santander Colombia beatriz@uis.edu.co

**Keywords:** nursing process, standardized nursing terminology, nursing methodology research, health knowledge, attitudes, practice, proceso de enfermería, terminología normalizada de enfermería, investigación metodológica en enfermería, conocimientos, actitudes y práctica en salud., processo de enfermagem, terminologia padronizada em enfermagem, pesquisa metodológica em enfermagem, conhecimentos, atitudes e prática em saúde.

## Abstract

**Objective:**

To analyze the literature available on the psychometric properties of the instruments to measure knowledge, attitudes, and practices of the nursing care process.

**Methods:**

This was a narrative-type review conducted by following the recommendations of the PRISMA declaration. The search strategy was executed in two stages; through the search in databases by two reviewers and - thereafter - three reviewers identified independently the studies and evaluated the methodological quality of the measurement instruments by using the COnsensus-based Standards for the selection of health Measurement INstruments (COSMIN) property checklist boxes.

**Results:**

Of 71 studies identified for the full-text review, only seven complied with the inclusion criteria that represent four instruments (Q-DIO, D-CATCH, NP-CDSS, PNP). It was found that the instruments continue in their validation and appropriation processes to reality in health services.

**Conclusion:**

In spite of the evident evolution of the instruments to evaluate the implementation of the nursing care process, the need is still valid for an instrument that measures aspects of knowledge, attitudes, and practices in every stage of the process.

## Resumen

**Objetivo:**

Analizar la literatura disponible sobre las propiedades psicométricas de los instrumentos para medir Conocimientos, Actitudes y Prácticas del Proceso de Cuidado de Enfermería.

**Métodos:**

Revisión de tipo narrativa según las recomendaciones de la declaración PRISMA. La estrategia de búsqueda se realizó en dos etapas; a partir de la búsqueda en bases de datos por parte de 2 revisores y, posteriormente, tres revisores identificaron de forma independiente los estudios y evaluaron la calidad metodológica de los instrumentos de medición utilizando la COnsensus-based Standards for the selection of health Measurement INstruments (COSMIN).

**Resultados:**

De 71 estudios identificados para la revisión de texto completo, solo 7 cumplieron los criterios de inclusión que representan 4 instrumentos diferentes (Q-DIO, D-CATCH, NP-CDSS, PPE). Se encontró que los instrumentos continúan en procesos de validación y apropiación de los mismos a la realidad en los servicios de salud.

**Conclusión:**

A pesar de la evidente evolución de los instrumentos para evaluar la implementación del Proceso de Cuidado de Enfermería, aún sigue vigente la necesidad de un instrumento que mida los aspectos de Conocimientos, Actitudes y Prácticas en todas las etapas del proceso.

## Resumo

**Objetivo:**

Analisar a literatura disponível sobre as propriedades psicométricas dos instrumentos de medida de Conhecimentos, Atitudes e Práticas do Processo de Cuidar de Enfermagem.

**Métodos:**

Revisão narrativa realizada de acordo com as recomendações da declaração PRISMA. A estratégia de busca foi realizada em duas etapas; por meio da busca nas bases de dados CINAHL, MEDLINE, BVS e Google Scholar por 2 revisores e, posteriormente, três revisores identificaram os estudos de forma independente e avaliaram a qualidade metodológica dos instrumentos de medição usando a COnsensus-based Standards for the selection of health Measurement INstruments (COSMIN).

**Resultados:**

Dos 71 estudos identificados para revisão de texto completo, apenas 7 preencheram os critérios de inclusão representando 4 instrumentos diferentes (Q-DIO, D-CATCH, NP-CDSS, PPE). Constatou-se que os instrumentos continuam em processos de validação e apropriação dos mesmos à realidade nos serviços de saúde.

**Conclusão:**

Apesar da evidente evolução dos instrumentos de avaliação da implementação do Processo de Cuidar em Enfermagem, persiste a necessidade de um instrumento que mensure os aspectos de Conhecimento, Atitudes e Práticas em todas as etapas do processo

## Introduction

The nursing staff is the principal provider of patient care, responsible for continually identifying health problems and implement and adjust their interventions and that of other health professionals;(1) for this, it has its own tool, which requires technical-scientific knowledge that systematize care,(2) known as the nursing care process (NCP). Its registry in the clinical chart permits nurses to show the impact generated by their interventions, which demonstrates the importance of their professional role, as well as their autonomy and contribution within the health staff.(3)

Nursing records are considered a quality indicator in patient care, thereby, tools are required to evaluate the information registered by the nursing professionals, thus, implementation of the NNN (North American Nursing Diagnosis Association - NANDA-I, Nursing Interventions Classification - NIC, and Nursing Outcomes Classification - NOC) standardized language has permitted significantly to organize the documentation of nursing work, making the adequate registry of diagnoses improve the documentation of the evaluation, quality of the interventions, and results obtained.( 4)

Evidence shows that different factors(5-7) exist associated with the NCP application, which correspond to knowledge,(8) attitudes, and practices.(9-12) Knowledge(13) promotes the capacity of professional to remain open to using sources of information, making these significant and useful for the professional practice. Attitudes play an important role in implementing conducts; they permit explaining how a subject exposed to a stimulus adopts a given practice and not another, hence, the attitude toward the nursing care process is a primordial factor in its use.(14) Lastly, the practices or behaviors are observable actions by an individual in response to a stimulus; that is, these are the concrete aspect, the action.(15)

Due to the foregoing, different strategies have been undertaken to evaluate skills in applying the NCP, given that it would be related with the effectiveness of its interventions;(16) instruments exist that evaluate one or two or more parts, until evaluating all its components.(17)

Evolution in the development of the evaluation of the NCP quality has been carried out bearing in mind criteria included in the first instruments proposed by Ziegler in 1984 (Ziegler Criteria for Evaluating the Quality of the Nursing Process - ZCEQNP) and by Nordstrom and Gardulf in 1996 (NoGA), which centered on the structure of the documentation of the nursing process. Later, the importance was discovered of measuring the attitudes of nurses, creating the *Positions on Nursing Diagnosis* (PND) in 1992, developed by Lunney and Krenz.(18) Björvell, Thorell-Ekstrand & Wredling (2000), which identified the need to evaluate not only the existence of data, but also their qualitative aspects. Thus, they proposed Cat-ch-ing, responding to the new characteristics of the nursing exercise, at the time being a more-independent practice in which the documentation of care had to include not only the timely and precise registry of the medical and nursing interventions performed, but also the decision process, explaining and evaluating the nursing actions.(19) In 2007, Müller-Staub M et al., evidencing in their systematic reviews that no instrument existed to measure the NNN, and based on a modified drafting of the ZCEQNP and on the seven-point scale by Lunney, they created the Q-DIO.( 20)

From these instruments to evaluate the NCP, it is important to know the type of psychometric properties evaluated and the methodological strategies used for their validation, from the simplest validity to evaluate, the apparent validity, to the most complex, validation of criterion and sensitivity to change. Different methods exist for such as of two large paradigms,(21) the classical theory of the test and the response theory to the item; the latter with some advantages over the other;(22) among those advantages, estimation is highlighted of statistics for the items and for the individuals, establishing the difficulty of the items and the ability of the individuals. Another advantage, in theory, is the invariability of the instrument’s parameters when calculated in groups of different abilities, making the independent estimations of the sample used comparable.

Moreover, in NCP implementation in the practice, instruments to measure its quality have been modified - responding to the challenges represented by each progress in the nursing records. This is how today, in the search of the use of electronic records throughout the world, initiatives of tools emerge that bear in mind the nurses’ practices, knowledge, and attitudes.

The aforementioned evidences that existing instruments to evaluate knowledge, attitudes, and practices (KAP) of the NCP report variability in their use over time, as well as in their validation process. Bearing in mind that validation of the instruments (face, content, construct, criterion, internal consistency, reproducibility, and sensitivity), permits establishing their reliability and reproducibility, whether for measurements at a given moment or for comparisons before and after applying interventions to determine their effectiveness or efficacy. In the health area,(23) the importance is highlighted of carrying out these processes and, finally, obtaining validated instruments to measure phenomena, given that often these are subjective phenomena.

Considering that a narrative review permits the objective evaluation of the characteristics of the instruments and, thus, identifies the most adequate for their use, the objective of this study was to describe the state-of-the-art of the instruments to measure KAP of the NCP and their psychometric properties.

## Methods

This narrative-type review was carried out by following the recommendations by the PRISMA declaration,(24) which has 27 items and a four-step flow diagram adapted to the literature search methodology and the selection of primary studies to be included in the synthesis of the evidence.

The search strategy was conducted in two stages; the first part started through the search by two reviewers in the CINAHL, MEDLINE, and BVS databases and in Google Scholar, guided under the question “Which instruments exist in the literature to measure knowledge, attitudes and practices related with the nursing process or the NNN standardized languages?, formulated from the P: patient or problem, I: intervention, C: compared with, O: Outcomes -results (PICO) question; using synonyms and MeSH term, thus: **P**: Nurse OR Registered Nurses OR Nursing; **I:** Surveys and Questionnaires AND Knowledge, Attitudes, Practice OR Attitude OR Practice OR Knowledge (MeSH) AND Nursing Records (MeSH); **C**: Does not apply; **O**: Nursing process (MeSH) OR Nursing diagnosis (MeSH) OR NANDA AND NIC AND NOC OR Nursing interventions OR Nursing outcomes OR Standardized Nursing Terminology (MeSH) OR Standardized nursing languages OR Standardized Nursing Data OR Nursing Diagnosis/standards OR Nursing Records/standards.

For this search, the limits were publications from 2010 to 2020, in English, Spanish, or Portuguese on studies conducted in humans.

The following shows an example of the search strategy in PubMed: *(((((Nurse) OR Nursing) OR Registered Nurses)) AND (((((((Surveys and Questionnaires)) AND Knowledge, Attitudes, Practice) OR Attitude) OR Practice) OR Knowledge) AND Nursing Records [MeSH Terms])) AND ((((((((((((Nursing process [MeSH Terms]) OR Nursing diagnosis [MeSH Terms]) OR NANDA) AND NOC) AND NIC) OR Nursing interventions) OR Nursing outcomes) OR Standardized Nursing Terminology [MeSH Terms]) OR Standardized nursing languages) OR Standardized Nursing Data) OR Nursing Diagnosis/standards) OR Nursing Records/standards)*

Upon ending this first stage, 16 instruments were identified, responding to the research question posed; this input gave continuity to the second stage that included a third reviewer. Each reviewer conducted an independent search, using the 16 names as search terms in the CINAHL, MEDLINE, and BVS databases and in the Google Scholar search engine. When necessary, the instrument’s authors were contacted to find articles that described clearly the evaluation of the psychometric properties of each instrument. This second stage was performed from March to May 2020, following the same limits already described.

Inclusion criteria. Studies were selected that conducted evaluation of psychometric properties to measurement instruments for: knowledge, attitudes, or practices related with the nursing process.

Exclusion criteria. The work excluded articles that did not completely describe the validation process, as well as those about instruments to which there was no access. It also excluded conference abstracts and case reports

Article selection. Selection of the documents was based on the agreement between the research question and the title/abstract, recovering the full texts to re-evaluate them according with the inclusion criteria; this process was carried out independently and in standardized manner by three reviewers. Each reviewer, after reading the full text for each article, filled out a sheet with the following items: name of the article, year, authors, complete description of the validation process, name of the instrument, and dimension it evaluates (knowledge, attitudes, or practices).

Thereafter, bearing in mind the name of each instrument, the search was conducted for it to identify author, creation data, name, language, number of items, form of scoring. From this sheet, consensus was reached among the reviewers to establish the articles to analyze. Said consensus was reached simultaneously through virtual meetings to carry out the discussion and analysis of each article

Data extraction. The study followed the recommendations of the COSMIN (25) tool’s manual for risk of bias. This table was filled out in Excel by a researcher and verified by another researcher. Data were extracted on the design, purpose, population, measurement instrument, properties of the instrument, author, year of publication, statistical tests, and statistical results of each study.

Evaluation of the quality of the articles. The methodological quality of the studies included was assessed through adjusting the COSMIN risk-of-bias control list,^26^ constructing an Excel spreadsheet for each article, each including 116 items, divided into the following sections: instrument development, content validity, structural validity, internal consistency, transcultural validity, reliability, measurement error, validity of criterion, hypothesis validity and response capacity tests. Each item was written in question form with the following response options: very well, adequate, doubtful, inadequate, or does not apply.

To respond to each item, virtual meetings were conducted with the presence of three reviewers, who verified each question in the full text, and in consensus the item evaluated was scored; when the question required it, the necessary literature search was carried out to respond to the item.

The final score of the methodological quality of each article was assigned bearing in mind the indication provided by the tool, that is, the article’s overall score corresponded to the lowest score found in any item.

In the search aimed at this review, the sample of interest was defined as the nurses’ records or the nurses who had completed an instrument (independent variable) to measure the practice, knowledge, or attitudes of applying the nursing process (the result or the dependent variable).

Psychometric properties. Upon defining the articles de mayor relevance that complied with the selection criteria and according with the COSMIN guide,(26) the study described the data related with internal consistency, reproducibility, face validity, content validity, construct validity, criterion validity, reproducibility, and sensitivity to change evaluated by each study. This narrative review was carried out within the frame of the research project "Effect of a formation program to implement the nursing process in a tier III health care institution" funded by the Vice-rectory of Research and Extension, Code No.2450 from Universidad Industrial de Santander and which was approved by the ethics committee in the Faculty of Health at Universidad Industrial de Santander.

## Results

In the search of the CINAHL, MEDLINE, and BVS databases and search in other sources (through bibliography references and Google Scholar) 11,288 articles were found. After adjusting the duplicates, 6,308 articles remained; of these, 2,297 were eliminated by applying search limits (publications from 2010 to 2020, in English, Spanish, or Portuguese). Thereafter, the second stage of the search was begun by name of instrument, which identified 150 articles and which were added to the main search.

**Figure 1:**
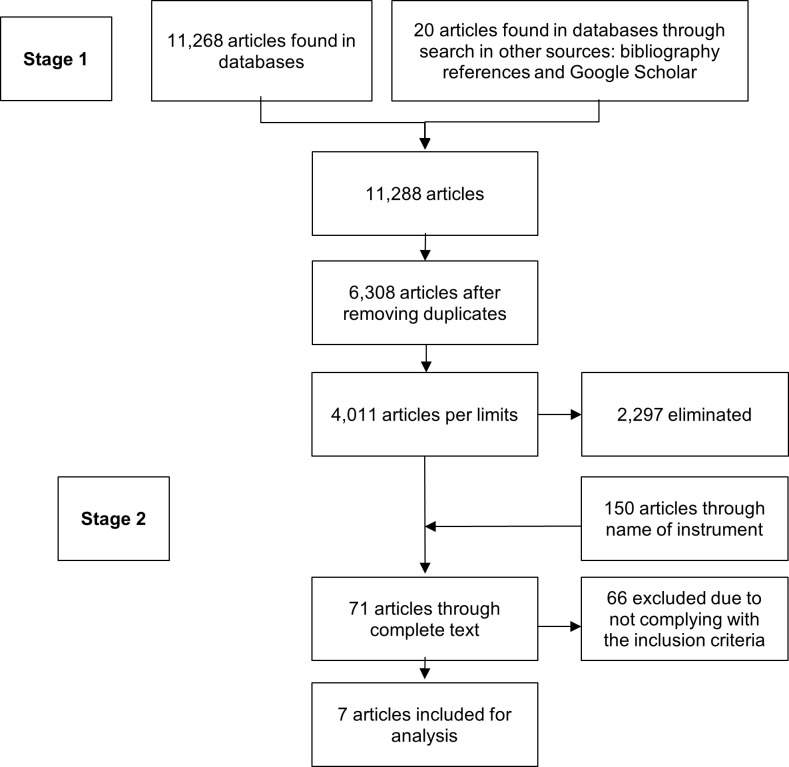
Flowchart of the article search and selection

Consecutively, with 4,161 articles, their review was started through title/abstract from which 4,090 articles were discarded due to not coinciding with the search objective and not having full text. A critical reading was performed of the 71 articles remaining, with application of the inclusion criteria and eliminating 16 because of no access to the instrument evaluated, 18 because they did not describe the complete evaluation process, 25 for not evaluating the complete nursing process and, finally, seven articles were selected for review (Figure 1).

Table 1 describes the instruments found in each article. In total, it was possible to identify four instruments: NP-CDSS, Q-DIO, D-CATCH, and PNP; all with a different scoring methodology. With respect to origin, only the PNP is of Latin American origin, against the rest that are of European origin. It may also be noted that the Q-DIO and D-CATCH have been adapted into another language, Portuguese and Italian, respectively. Regarding the way of evaluating, only the NP-CDSS does it qualitatively, while the rest do it quantitatively. In relation to the component, only the PNP evaluates attitudes regarding the NCP; the rest evaluate the practices, and none evaluates knowledge or the three components simultaneously.

**Table 1:** Description of instruments

Müller-Staub *et al.,* (2016). The Netherlands.(27)	Nursing Process-Clinical Decision Support System Standard Development - NP-CDSS
	*Description:* 15 items evaluate NCP as central piece of information and nursing documentation; 10 items evaluate the use of data recovery and additional evaluations.
	Evaluates the NCP practice qualitatively.
	*Items*: 25
da Costa Linch *et al.,* (2012)*; Brazil*(28)	Quality of Diagnoses, Interventions and Outcomes (Q-DIO - Portuguese version)
	*Description:* 11 items evaluate nursing diagnoses as process, 8 items evaluate nursing diagnoses as product, 3 items evaluate nursing interventions and 7 items evaluate nursing results. Each item is scored with a 3-point Likert-type scale; and evaluates the quality of nursing diagnoses and determines the sensitivity of the interventions and results of patient care.
	*Items:* 29.
	*Component evaluated:* Practice.
*Müller-Staub et al., (2010); Switzerland.*( 29)	Quality of Diagnoses, Interventions and Outcomes (Q-DIO)
	*Description:* 11 items evaluate nursing diagnoses as process (3-point Likert-type scale), 8 items evaluate nursing diagnoses as product (5-point Likert-type scale), 3 items evaluate nursing interventions (5-point Likert-type scale) and 7 items evaluate nursing results (5-point Likert-type scale). Evaluates quality of nursing diagnoses and determines the sensitivity of the interventions and results of patient care.
	*Items:* 29.
	*Component evaluated:* Practice.
da Costa Linch *et al.,* (2015); Brazil.( 30)	Quality of Diagnoses, Interventions and Outcomes Q-DIO - Portuguese version
	*Description:* 11 items evaluate nursing diagnoses as process. 8 items evaluate nursing diagnoses as product, 3 items evaluate nursing interventions and 7 items evaluate nursing results. Each item is scored with a 3-point Likert-type scale; and evaluates the quality of nursing diagnoses and determines the sensitivity of the interventions and results of patient care.
	*Items:* 29.
	*Component evaluated:* Practice.
D'Agostino *et al.,* (2015); Italy.( 31)	D-Catch Italian version
	*Description:* 1 item evaluates the structure of the record according with the NCP, 1 item appraises data on admission, 1 item evaluates the nursing diagnoses with PES structure, 1 item evaluates the interventions (related with the diagnosis), 1 item evaluates the follow up and evaluates the results (related with the diagnosis) and 1 item evaluates the legibility of the documentation. Each item is scored with a 3-point Likert-type scale; and evaluates the precision of the nursing documentation in hospitals.
	*Items:* 3*.*
	*Component evaluated:* Practice.
Guedes *et al.,* (2013); Brazil.( 32)	Positions on the nursing process - PNP
	*Description:* The items represent adjectives evaluated with a 7-point Likert-type scale. Evaluates perception regarding the NCP.
	*Items:* 20.
	*Component evaluated:* Attitudes.

Table 2 shows that only two studies(10,27) reported the instrument’s creation process, identifying that Müller(27) did not report if the problems identified in the first evaluation by experts were addressed or if the instrument was again tested with these improvements.

With respect to content validity, four studies(10,27,28, 31) show evaluation of this aspect; only the D-CATH original (29) reported numerical value with K > 0.62; phase validity was conducted by an average of eight experts (NPCDSS: 8, Q-DIO Portuguese: 9, D-CATCH Italy: 4, D-CATCH original: 12). It was found that in most of the studies the number of experts was < 30; participation by at least two or more researchers was not clearly identified, nor was clarity found on the method and approach to analyze the evaluation data.( 33) To evaluate the construct validity, the studies were based on the classical theory, using the most adequate statistical methods for the case: the confirmatory factorial analysis and exploratory factorial analysis. It was established that, overall, all the studies used a sample size classified as very good according to COSMIN(34-39) (seven times the number of items and > 100), as reported by Guedes(32) who proved that the PNP measures the three dimensions proposed in its hypothesis.

Internal consistency was reported by six of seven studies(10,28-32) with Cronbach’s alpha values ranging between 0.70 and 0.99, evidencing that, generally, these have good reliability; it must be highlighted that in the evaluation of the methodological quality in the D-Cath original and D-Cath Italy, this value was not calculated in each subscale.

The COSMIN checklist includes transcultural validity, convergent validity, and discriminatory validity carried out in the study by Linch(30), which identified lack of clarity in reporting similar characteristics of the groups (except for the study variable), as well as the use of a statistical method (***p*** ) poorly adequate to measure the relations.

It was found that in the Q-DIO original study,(29) reproducibility was evaluated with Pearson’s and Spearman’s correlation coefficients, which ignore the dependence of the measurements; on the contrary, the Portuguese version was evaluated with the ICC, the most-adequate statistical method to evaluate reproducibility. Intra- and inter-evaluator and agreement correlation values were reported in four studies. Specifically, in the study by Linch(30), the Q-DIO reported deficient ICC, given that the instrument was more reproducible where the record was electronic without process, followed by electronic with process and poorly reproducible in centers where records were handwritten and without standardized language.

No study reported validity of criterion, error measurement, or response capacity.

Among the limitations and recommendations described by each study, performance is highlighted of validation studies with a broader sample, which include settings different from the hospital, as well as the application of transcultural adaptation processes to test them at international level. Likewise, it is recommended to conduct elaboration processes of operational definitions for the items of the instruments to facilitate standardization of their application. Finally, the study highlights the usefulness of nursing records as important source of data for research.

**Table 2:** Analysis of the quality of the evaluation of the psychometric properties of the instruments

Name of the instrument - Version	n	Analysis unit	Design proces	Content validity	Construct validity	Internal consistency	Transcultural validity	Reproducibility	Error measurement	Validity of criterion	Convergent validity and or groups	Response capacity
NP-CDSS original (27)	27	Clinical nurses	-	+	N/A	N/A	N/A	N/A	N/A	N/A	N/A	N/A
NP-CDSS original (27)	8	Experts	-	+	N/A	N/A	N/A	N/A	N/A	N/A	N/A	N/A
Q-DIO portugués(28)	40	Registries	N/A	+	N/A	++	N/A	N/A	N/A	N/A	N/A	N/A
Q-DIO original (29)	60	Registries	N/A	N/A	N/A	+++	N/A	-	N/A	N/A	N/A	N/A
Q-DIO portugués(30)	168	Registries	N/A	N/A	N/A	+++	+	+	N/A	N/A	Convergent: + Groups: +	N/A
D-Catch original (10)	245	Registries	-	+	+++	+	N/A	N/A	N/A	N/A	N/A	N/A
D-Catch Italy (31)	250	Registries	N/A	+	+++	+	N/A	N/A	N/A	N/A	N/A	N/A
D-Catch Italy (31)	40	Pilot Regis-tries	N/A	+	+++	+	N/A	N/A	N/A	N/A	N/A	N/A
PPE original ^32^	632	Nurses	N/A	N/A	+++	+++	N/A	N/A	N/A	N/A	N/A	N/A
PPE original ^32^	973	Aides	N/A	N/A	+++	+++	N/A	N/A	N/A	N/A	N/A	N/A

Very well (+++), Adequate (++), Doubtful (+), Inadequate (-), N (does not apply)

## Discussion

Although instruments to evaluate the nursing process started being developed since 1994(33), this review permitted evidencing that the instruments that have been adapted most transculturally, used and validated, have been the Q-DIO and D-CATCH, which evaluate NCP application in the practice, through the review of nursing records; however, these instruments have measurements for the dimension of Practices or Behaviors, without finding measurement for knowledge and attitudes. The difference lies in that the first evaluates quantitatively its items and the latter does so quantitatively and qualitatively; in turn, the distribution of the questions differs in the amount (29 and 6, respectively) and the orientation of their evaluation. The foregoing contrasts with instruments, like the Application of the Nursing Process in Health Institutions (APEIS, for the term in Spanish),(36) found in the literature search and which has items to evaluate the three KAP dimensions, reporting adequate internal consistency with Cronbach’s alpha of 0.854; nevertheless, the article evaluated reports no description of the methodological process and analysis of the psychometric properties; hence, it did not comply with criteria to be included in the score of methodological quality of this systematic review.

Another important aspect to highlight is that only one instrument included in this review was created and evaluated for Latin America, titled Positions on the Nursing Process (PNP) original(32) in Portuguese that measures perceptions of the Nursing process on a self-filled form, which together with other instruments, like APEIS(40) and the instrument used for the situational diagnosis of the systematization of nursing care in a basic health unit, as self-filled instruments, were not included in this review because no report was found of the evaluation process of psychometric properties. The D-CATH(10) is proposed as another adequate instrument to evaluate the quality of the records; given that this review found no articles that showed its use in Latin American, transcultural adaptation and evaluation of psychometric properties in this context would be important.

To minimize biased or undue results that lead to erroneous conclusions in studies,(41) emphasize that every instrument must be evaluated and validated prior to being used; according to them, it was possible to observe that, although the face validity reported by all the instruments in this review was relevant, the content validity was not reported in the same manner, which would give more support to the instrument’s conceptual description. Moreover, an instrument with construct validity will permit(42) determining the integration of the conceptual abstraction for applicability; said estimation was performed on the Q-DIO, PNP and D-CATCH instruments (original version and Italian). Lastly, the validity of criterion that would permit approaching the praxis beyond the conceptualization was not reported in any of the instruments reviewed in this study. These types of studies should have the sample size, which must have a participant/item rate >10 and, in this review, four studies coincided with this sample.(43) The study by Paans(18) reports values that indicate good reproducibility and internal consistency, like psychometric properties of the Cat-ch-ing, QOD and Scale instruments for degree of accuracy in Nursing diagnoses, characteristics that coincide with the D-CATCH and Q-DIO v, formulated from those mentioned previously and evaluated since 2010.

None of the instruments reported measured in general the precision of the PE documentation in the electronic health records; based on that, in 2016 Müller developed the NP-CDSS standard,( 20) to which face and content validity tests were performed and given that it is in the initial stages, the possibility is contemplated of including it in future systematic reviews that evidence progress in its validation. The instruments continue in their process of validation and appropriation to the reality in the health services.

Among the limitations of validating the instruments analyzed, it is mostly found that the data collection was conducted retrospectively with the review of records made with an antiquity of two years, which can be interpreted as information bias, given that the recommendation(44) is for the instruments to be applied to the records in the least time possible after being written to permit clarifying the existence of data or their location and, thus, diminish this bias. Few studies describe the calculation of the sample size and another limitation evidenced was the stratification of the sample without considering it in the analysis; without evaluating if said stratification alters the results of the psychometric tests.(45)

## Conclusion

This review shows the progress and relevance in measuring content and construct validity by using the classical theory of psychometry, of instruments that strengthen the follow up of the application of the NCP in health institutions; but the need persists to conduct comparative studies of the instruments in practical contexts and in the electronic records of the NCP; as well as the use of theories of response to the item to measure the construct and criterion validity. In spite of the evident evolution of the instruments to evaluate the implementation of the NCP, there is still need for an instrument to measure the three KAP aspects in all the stages of the process, with the rigor of the validation and report of its psychometric properties, for its application in the practice.
